# Effect of Immersive Virtual Reality by a Computer Assisted Rehabilitation Environment (CAREN) in Juvenile Huntington’s Disease: A Case Report

**DOI:** 10.3390/medicina58070919

**Published:** 2022-07-11

**Authors:** Roberta Cellini, Giuseppe Paladina, Giacomo Mascaro, Maria Antonietta Lembo, Antonino Lombardo Facciale, Maria Cristina Ferrera, Bartolo Fonti, Luca Pergolizzi, Piero Buonasera, Placido Bramanti, Emanuela Mazzon

**Affiliations:** IRCCS Centro Neurolesi “Bonino Pulejo”, 98100 Messina, Italy; giuseppe.paladina@irccsme.it (G.P.); giacomo.mascaro@irccsme.it (G.M.); maria.lembo@irccsme.it (M.A.L.); antonino.lombardo@irccsme.it (A.L.F.); mariacristina.ferrera@irccsme.it (M.C.F.); bartolo.fonti@irccsme.it (B.F.); luca.pergolizzi@irccsme.it (L.P.); piero.buonasera@irccsme.it (P.B.); placido.bramanti@irccsme.it (P.B.); emanuela.mazzon@irccsme.it (E.M.)

**Keywords:** juvenile Huntington’s disease, CAREN, neurorehabilitation, virtual reality

## Abstract

Various studies have proven the utility of immersive virtual reality (VR) as a complementary approach to conventional neurorehabilitation therapy for improving neuromuscular and cognitive outcomes in several neurological diseases. We hereby report findings from a single-case experience of a 21-year-old woman affected by juvenile Huntington’s disease (HD) who underwent a targeted rehabilitative approach using an advanced Computer Assisted Rehabilitation Environment (CAREN) with a three sessions/week schedule for six months. At the end of the program, a manifested improvement was noticed in the Falls Efficacy Scale International score, in the Tinetti Scale, in the Berg Balance score and in the lower limb strength (MRC scale). Minor although tangible improvements were also noticed in some physical performance tests (10 m walking test, time up and go test). Findings reported, although preliminary, extend for the first time the usefulness of neurorehabilitation using innovative VR technologies also to juvenile HD, a condition for which common rehabilitation strategies bring only marginal physical benefits in the majority of cases. Future, controlled studies are awaited for generalizing these observations to larger populations and for clarifying whether such benefits may persist also in the long-term.

## 1. Introduction

Huntington’s disease (HD) is a rare, genetic disease with autosomal dominant inheritance leading to a progressive degeneration of nerve cells in the brain, which results in movement, cognitive and psychiatric disorders [1]. Although first symptoms manifest when people are in their 30s or 40s, juvenile forms with faster progression and worsen clinical manifestations are often described. Key features of HD encompass a wide spectrum of neuromuscular manifestations including involuntary jerking, writing movements, muscle rigidity or contracture, slow or abnormal eye movements, impaired gait, posture and balance and difficulty with speech or swallowing. Cognitive and psychiatric disorders are also not infrequently reported [2].

There are currently no pathogenic treatments for HD. Drug therapy somewhat improves movement impairment and psychiatric manifestations, while physical therapy may enhance strength but brings limited benefits on flexibility, balance and coordination. Hence, the overall impact of single or combined therapies results inadequate, particularly in juvenile forms [3].

In recent years, the employment of virtual reality (VR) has been proposed as a complementary approach to conventional therapy for improving physical and cognitive outcomes in various neurological disorders such as stroke-induced paralysis, multiple sclerosis, Parkinson’s disease, traumatic brain injuries and cognitive deficits [4,5,6,7].

In all these conditions, the simulated environment stimulates the executive functions, the visuo-spatial abilities, the attention and, no less important, the emotion and overall participation of the patient to the rehabilitation program. Hence, such an approach could theoretically be useful in HD subjects, in whom the scarce physical improvements driven by traditional treatments often translate into a lack of motivation and limited compliance to therapy prescriptions which, in turn, contribute to depression.

The immersion of the user into VR helps creating a sense of connectedness, which ameliorates the response to stimuli and the engagement to practicing with the required movements, thereby improving the overall adherence to physical treatments.

Yet, to the best of our knowledge, the potential usefulness of VR has never been tested in individuals with HD.

We hereby report, for the first time in the literature, a case of a young woman affected by HD with juvenile onset who underwent a targeted rehabilitative approach using an advanced Computer Assisted Rehabilitation Environment (CAREN) to ameliorate symptoms and improve motor recovery.

## 2. Case Description

L.P. was a 21-year-old woman affected by juvenile HD and followed in an outpatient setting at the IRCCS Neurolesi “Bonino-Pulejo” in Messina, Italy. Diagnosis was made 4 years before starting the VR program, based on the presence of mood and motor disturbances and evident family inheritance (the father was affected by HD) and subsequently confirmed by genetic test. She was poorly responsive to antidepressant and anxiolytics.

At the initial neurological evaluation, the patient presented a moderate strength deficit, particularly evident at the left upper limb level, with slight dysmetria in the Finger-to-Nose-Test and difficulty in motor coordination during the execution of complex movements. A stenic deficit was detectable in lower limbs, particularly on the left side, with limitation in the range of movements (ROM) of the hip and knees and with the presence of joint noises upon passive mobilization. Dysmetry in the Heel-to-Shin test was also present. Romberg′ s sign was positive with a tendency to retropulsion after pushing tests. The maintenance of a prolonged erect position was severely impaired due to the rapid appearance of general fatigue. Walking was possible without aids although difficult, particularly when performed on the heels. The patient needed help in carrying out the activities of daily living.

The patient was poorly compliant to standard physical therapy. Upon multidisciplinary consultation, the medical staff thus decided to start a VR rehabilitation program using an advanced Computer Assisted Rehabilitation Environment (CAREN), with the aim of improving postural activities and muscular endurance, facilitating strength recovery and achieving a global reorganization of motor activities, execution speed and motor coordination. Given the young age of the patient and the more evident impairment in lower limbs function which severely affected daily life and working activities, the VR treatment was particularly focused on strengthening the orthostatic isometric capacity and increasing resistance in standing position and during walking, in order to retard the appearance of tiredness and muscle fatigue.

**Figure 1:**
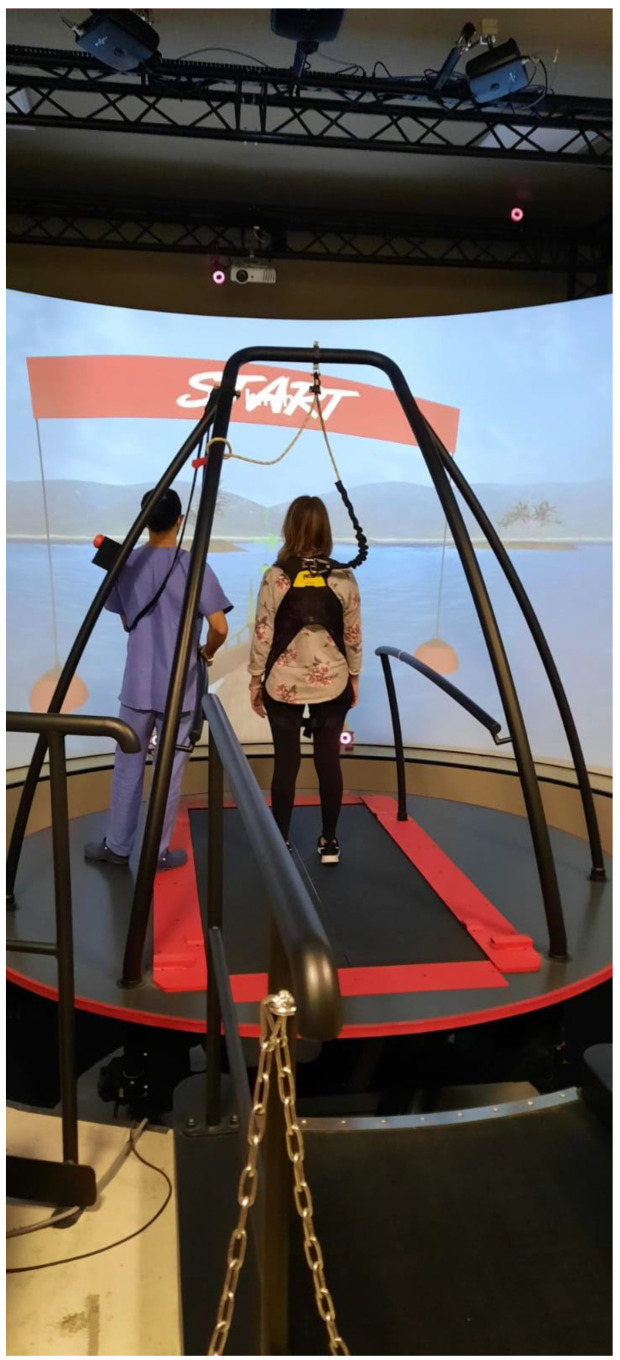
A screenshot of the patient undergoing a training session on the CAREN VR rehabilitation system.

The CAREN system (MOTEK Medical; Amsterdam, The Netherlands) consists of a motion capture system and a base driven by hydraulic and mechanical actuators (i.e., a 6 degrees of freedom motion platform and built-in instrumented treadmill). The device is equipped with 180 screen and varying degrees of VR immersion ranging from a flat video, dual-channel audio to a 360 surround sound dome enclosure. The platform used at our Centre also included sensory inputs such as visual, auditory, vestibular and tactile, allowing the operator to generate cognitive, visual and physical perturbations which forces the user to perform dynamic responses during the gait patterns (Figure 1).

During the experimental sessions, the patient was secured in a full body safety harness attached to an overhead truss, which allowed her to move freely on the treadmill. The rehabilitative program was realized in the immersive virtual environment by using six virtual scenarios:

1. MM Boat: The setting was a marine environment with buoys to avoid; the patient guided the boat with his body until the final goal.
2. Microbes: the setting was an environment with microbes to avoid and with different tasks.
3. Active Balance: a labyrinth in which the patient drove a red ball, moving the load up to the finish line.
4. Road encounters: A forest in which the patient walked or ran, with ascents or descents and was asked to strike at the same time some distractor elements (butterflies or birds).
5. Italian Alps: a country set in the Italian alps in which the patient walked, with ascents or descents; while walking, the patient was prompted to collect some ingredients to make a pizza.
6. Traffic jam: the setting was a crossroads with the patient driving a car.

The rehabilitation program was articulated in three sessions per week for six months, with each session lasting at least 45 min. Before starting the program and immediately after the last session, we employed the Falls Efficacy Scale International, the Tinetti Scale and the Berg Balance Scale to assess the patient’s balance and the Medical Research Council (MRC) scale, to estimate the muscular strength of upper and lower limbs. We also measured the overall physical performance by the 10-m walking test, the timed up and go test and the 6 min walking test. Additionally, an instrumental evaluation of balance control was made by a static balance analysis calculating the average of the contact forces of the foot on the ground (ZEBRIS device; ZEBRIS Medical GmbH, Germany).

The patient completed the VR rehabilitation program without experiencing any adverse event, such as falls, neurovegetative syndromes or musculoskeletal injuries.

Upon treatment completion, the patient experienced an increase in muscle strength, particularly at the lower limbs level, with a following improvement in the coordination deficit even while executing more complex movements. The joint limitations regressed thereby ameliorating the overall stability. An increased resistance in standing position and endurance while walking was also documented, with a delayed onset of muscle fatigue which allowed the patient to be more independent during daily life activities.

**Figure 2:**
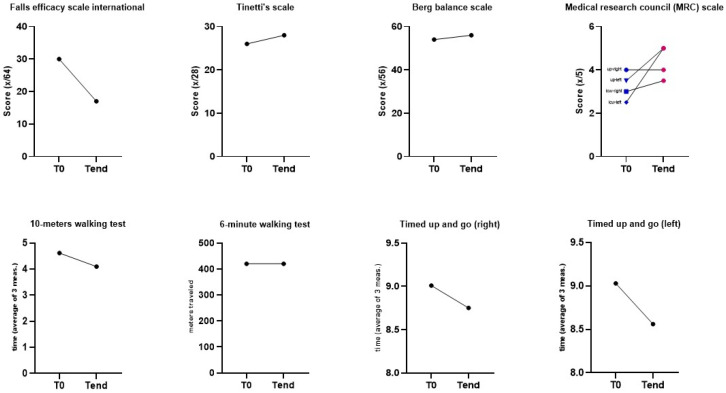
Changes in main scales and physical performance tests from baseline (T0) to the end of the established VR rehabilitation program (Tend). MRC scale: blue circle is the up-right limbs baseline value; blue triangle is the up-left limbs baseline value in; blue square is the low-right limbs baseline value; blue rhombus is the low-left limbs baseline value.

Figure 2 summarizes the main changes reported in the evaluation scales and physical performance tests from baseline to the end of the established VR rehabilitation program.

Briefly, a manifested improvement was noticed in the Falls Efficacy Scale International score (from 30/64 to 17/64, which coincides with a documented reduction in the fear of falling), in the Tinetti Scale (from 26–28 to 28/28, which stands for an improvement in balance and walking) and in the Berg Balance score (increased from 54/56 to 56/56, indicating an overall improvement in balance control).

No changes were noticed in the MRC Scale for the right upper limb strength (4/5 before and after the VR treatment) while only a marginal improvement was obtained in the left upper limb strength (from 3/5 to 3+/5 at the end of treatment). Conversely, a greater improvement was reported for the lower limb strength, with the right limb score increasing from 3+/5 to 5/5 and the left limb score increasing from 3/5 to 5/5.

Physical performance tests documented a slight reduction in the time to complete the 10-m walking test, which decreased from 4.62 to 4.10 s, and a similar reduction in completing the “time up and go” which time decreased from 9.03 to 8.56 s in turning left and from 9.01 to 8.75 in turning right.

Finally, controversial findings were obtained at the static balance analysis evaluation with both eyes closed or open, showing an improvement in the average strength on the hind-foot but an apparent worsening on the fore-foot (Table 1).

## 3. Discussion

In this case study, we have reported preliminary evidence of how a VR rehabilitation program by CAREN, an advanced, computer-assisted system with different engaging immersive scenarios, may contribute improving motor impairment, strength and balance in a patient affected by juvenile HD.

Juvenile HD encompasses more aggressive forms of HD, with earlier and more severe symptoms onset and for which common rehabilitation programs, alone or complemented by symptomatic drug treatment, bring only marginal physical benefits in the majority of cases [3].

In last years, various evidence has accrued demonstrating the potential usefulness of innovative technologies, such as immersive VR by CAREN, in addition to conventional physical therapy, to improve balance, stability and strength in patients with severe motor impairment due to neuromuscular disorders or even after transtibial amputation [5,8].

As a matter of example, individuals with Parkinson’s disease experienced a significant improvement in walking speed and walking stability with wider and longer steps after a standardized, long-term rehabilitation program by CAREN [9,10].

Significant benefits were also reported for gait speed and weight shift in adult subjects with traumatic brain injury and vestibular dysfunction [11] and comparable effects have been described also in younger individuals with similar brain injuries [12]. No less important, rehabilitation by the CAREN system has been demonstrated to be effective in ameliorating balance and in reducing the fear of falling in patients affected by multiple sclerosis [13], a finding which pairs well with some of the improvements observed in our young HD patient.

The exact mechanism through which VR may improve neuromuscular function remains questioned.

VR is alike to stimulate specific brain regions, likely including the mirror neuron system, which are responsible of motor planning and learning, thus improving the overall motor performance [6].

Beside this, an advanced VR rehabilitation program by the CAREN system holds the advantage of supplying immediate feedback as to performance, thus assisting with the learning of new motor strategies of movement with an acceptable approximation of the real world. Additionally, the diverse settings and game situations placed may increase compliance, retention and user satisfaction, being thus potentially beneficial for long-term effectiveness of rehabilitation programs. In our young HD patient, a structured, immersive VR program was effective to enhance motivation, engagement, commitment and the overall adherence to the rehabilitation program which, at the end, produced a tangible improvement in motor abilities, muscle strength and lower-limb coordination. Importantly, such benefits ended up with improving the overall patient autonomy by conveying a significant regression in joint limitations, a delayed onset of muscle fatigue with a following increased resistance in standing position and endurance while walking. This allowed the patient, at the end, to improve her autonomy in daily life and working activities, as initially desired.

We acknowledge that findings from a single-case experience, although promising, should only be considered as preliminary as it must be pondered against potential selection bias, confounding by indication and over-estimation of effects. Nevertheless, the benefits observed in strength, physical performance and fear of falling, to our opinion, deserve further in-depth investigations by structured, controlled studies in larger cohorts. These latter may also help evaluating the impact of such an immersive and engaging approach on the patient’s quality of life, therapeutic adherence and overall motivation.

## 4. Conclusions

Findings from this single-patient experience support the hypothesis that neurorehabilitation using innovative VR technologies can contribute improving commitment and motivation during the rehabilitation process, also bringing possible positive effects on various functional outcomes also in patients with juvenile forms of Huntington’s disease. Future studies are eagerly awaited for a possible generalization of these preliminary findings and for clarifying whether such benefits may persist also in the long-term.

## Figures and Tables

**Table 1:** Balance static analysis with open and closed eyes before and after the CAREN VR rehabilitation program.

Static Analysis with Open Eyes		
Test	Before	After
average left forefoot strength	38%	33%
average right forefoot strength	49%	42%
average left hindfoot strength	62%	67%
average right hindfoot strength	51%	58%
**Static Analysis with Close Eyes**		
**Test**	**Before**	**After**
average left forefoot strength	34%	31%
average right forefoot strength	36%	31%
average left hindfoot strength	66%	69%
average right hindfoot strength	64%	69%

## Data Availability

Not applicable.
